# Squamosamide Derivative FLZ Protects Pancreatic *β*-Cells from Glucotoxicity by Stimulating Akt-FOXO1 Pathway

**DOI:** 10.1155/2015/803986

**Published:** 2015-06-17

**Authors:** Xiangchen Kong, Longmei Zhang, Xianxin Hua, Xiaosong Ma

**Affiliations:** ^1^Diabetes Center, Shenzhen University, Shenzhen 518060, China; ^2^University of Pennsylvania Perelman School of Medicine, Philadelphia, PA 19104, USA

## Abstract

Chronic hyperglycemia increases apoptosis and reduces glucose-stimulated insulin secretion. Although protective agents have been searched extensively, none has been found so far. Here we tested FLZ, a synthetic derivative of squamosamide from a Chinese herb, as a potential candidate for antiglucotoxicity in INS-1E cells and mouse islets. Chronic culture of *β*-cells in 30 mM glucose caused progressive reduction of cell viability, accompanied with increased apoptosis and reduced insulin secretion. These effects on apoptosis and insulin were reversed by FLZ in a dose-dependent manner. FLZ treatment also increased forkhead box O1 protein phosphorylation and reduced its nuclear location. On the contrary, FLZ increased pancreatic and duodenal homeobox-1 expression and its nuclear localization, an effect mediated by increased p-Akt. Consistently, Akt selective inhibitor MK-2206 completely abolished antiglucotoxicity effect of FLZ. Furthermore, FLZ treatment increased cytosolic ATP/ADP ratio. Taken together, our results suggest that FLZ could be a potential therapeutic agent to treat the hyperglycemia-induced *β*-cell failure.

## 1. Introduction

Chronic exposure of pancreatic *β*-cells to high glucose causes pancreatic *β*-cell failure characterized with an increase in apoptosis and a marked deficit of glucose-stimulated insulin secretion (GSIS) [1], termed glucotoxicity. Based on the failure of *β*-cell on the pathogenesis of type 2 diabetes, it is important to prevent and/or reverse hyperglycemia-impaired *β*-cell functions.

FLZ is a synthetic derivative (molecular weight of 458.2) of squamosamide and is formulated as N-[2-(4-hydroxy-phenyl)-ethyl]-2-(2,5-dimethoxy-phenyl)-3-(3-methoxy-4-hydroxy-p-henyl)-acrylamide. It has been found that FLZ has prominent antiapoptotic effect on dopaminergic neurons that are exposed to MPP^+^ [2, 3] and 6-hydroxydopamine (6-OHDA) [4, 5] and also exerts neuroprotective effects on Parkinson's disease models [6]. The application for clinical trials of FLZ is under review by China Food and Drug Administration. Exactly how FLZ exerts its antiapoptotic effects remains unestablished but the effect may involve reducing mitochondrial oxidative stress [6]. Given that mitochondrial oxidative damage plays a key role in pancreatic *β*-cell failure in the pathogenesis of type 2 diabetes and given that mitochondria-targeted antioxidants protect pancreatic *β*-cells against oxidative stress under glucotoxic conditions [7], we therefore explored whether FLZ would prevent pancreatic *β*-cell glucotoxicity in this study.

## 2. Materials and Methods

### 2.1. Reagents and Antibodies

FLZ, a white powder with 99% purity, was kindly provided by Professor Dan Zhang from Institute of Materia Medica, Chinese Academy of Medical Sciences, and Peking Union Medical College. 3-(4,5-Dimethylthiazol-2-yl)-2,5-diphenyltetrazolium bromide (MTT), Hoechst 33342 (HO), propidium iodide (PI), sodium pyruvate, glutamine, and beta-mercaptoethanol were purchased from Sigma (St. Louis, MO, USA). MK-2206 was purchased from Selleck Chemicals (Houston, TX, USA). Collagenase P was provided by Roche Molecular Biochemicals (Indianapolis, IN, USA). PDX-1, Akt, Lamin B, and *β*-actin antibodies were purchased from Santa Cruz Biotechnology (CA, USA). p-Akt (Ser473), p-FOXO1 (Ser256), and FOXO1 antibodies were obtained from Cell Signaling (Danvers, MA, USA). Goat and rabbit secondary antibodies were product of Sigma (St. Louis, MO, USA). Rat and mouse Insulin Ultrasensitive ELISA kit were purchased from ALPCO Diagnostics (Salem, NH, USA). Nuclear-cytosol extraction kit was purchased from Applygen Technologies (Beijing, China). ADP/ATP assay kit was provided by BioVision (Mountain View, CA, USA).

### 2.2. Cell Culture

Rat pancreatic INS-1E cells were kindly provided by Professor Yong Liu from Shanghai Institute for Nutritional Sciences of Chinese Academy of Sciences. INS-1E cells between passages 60–90 were cultured in RPMI1640 medium, as reported previously [8].

### 2.3. Islet Isolation

Female C57BL/6 mice (4 w) were purchased from the experimental animal centre of Guangdong Academy of Medical Science (Guangzhou, China). The mice were sacrificed by cervical dislocation. Pancreatic islets were isolated and cultured using the methods reported previously [8]. Use and care of mice for the experiments were approved by the ethical committee overseeing research involving animals at Shenzhen University.

### 2.4. Cell Viability

Cell viability was assessed with MTT assay. The assay is based on the ability of living cells to convert MTT into insoluble formazan. Thus, the amount of produced formazan is proportional to the number of living cells. In detail, INS-1E cells were seeded in 96-well plates and cultured in 11.1 mM glucose (G11.1) or 30 mM glucose (G30) in the presence or absence of different concentration of FLZ and with or without 50 nM MK-2206 for 96 h. Thereafter, 100 *μ*L of 0.5 mg/mL MTT was added to each well and incubated at 37°C for 4 h. The resulting formazan was dissolved in 100 *μ*L DMSO. Samples were read at 570 nm using a microplate reader (BioTek Epoch, USA).

### 2.5. Apoptosis Assay

Apoptotic assay was carried out using Hoechst 33342 (HO) and propidium iodide (PI) staining method and counting cell numbers under a fluorescent microscope. In brief, after treating cells with 30 mM glucose for 96 h, the cells were incubated with 20 *μ*g/mL HO and 10 *μ*g/mL PI at 37°C, 5% CO_2_ for 15 min. Then the cells were washed once with PBS and the number of apoptotic cells was counted under an inverted fluorescent microscope. A minimum of 500 cells was counted for each plate under randomized conditions.

### 2.6. GSIS and Insulin Content Assay

INS-1E cells or mouse islets were seeded in 24-well plates and were cultured in 5.5 mM glucose (G5.5) or 30 mM glucose (G30) in the absence or presence of 10 *μ*M FLZ and with or without 50 nM MK-2206 for 72 h. Then insulin release from mouse islets and INS-1E cells was assayed as published [8]. In brief, the cells were preincubated in 1 mL Krebs Ringer bicarbonate (KRB) buffer for 1 h and then were stimulated with 1 mL KRB buffer containing 16.8 mM glucose for 30 min. The supernatants were collected for measurements of secreted insulin using Insulin Ultrasensitive ELISA kit. For INS-1E cells, the attached cells were harvested for determination of total cellular protein content. Insulin levels (ng/mL) were normalized against total cellular protein content in mg. For mouse islets, insulin levels (ng/mL) were normalized against islet number. For insulin content assay, after treatment with FLZ in the presence or absence of MK-2206 for 72 h, the cells were lysed with RIPA lysis buffer. The insulin levels in the cell lysates were determined with Insulin Ultrasensitive ELISA kit. Insulin levels (ng/mL) were normalized against total cellular protein content in mg.

### 2.7. Western Blotting

For extraction of total protein, cell pellets were incubated in RIPA lysis buffer supplemented with 1 mM protease inhibitor cocktail for 30 minutes on ice, followed by centrifugation at 14,000 g for 10 minutes at 4°C. Cytosolic and nuclear protein were prepared using a nuclear-cytosol extraction kit according to the manufacturer's protocol. Cell lysates were separated using SDS-PAGE gels and transferred onto PVDF membrane. The membranes were immunoblotted with respective primary antibodies overnight at 4°C and then incubated with horseradish peroxidase-conjugated secondary antibody for 2 h at room temperature. The immunoreactive bands were visualized by the KODAK Image Station 4000MM PRO imaging system and the densities of the bands were determined using Gel-Pro Analyzer 4.0 software and normalized against the level of *β*-actin or Lamin B.

### 2.8. ATP/ADP Assay

INS-1E cells were seeded in 96-well plates and cultured in 5.5 mM or 30 mM glucose medium in the presence or absence of 10 *µ*M FLZ for 72 h. After preincubation in 1 mL KRB buffer for 1 hour at 37°C, the cells were stimulated with KRB buffer containing 16.8 mM glucose for 30 min. Then ATP and ADP were measured using BioVision ADP/ATP Ratio Assay Kit according to the manufacturer's protocol.

### 2.9. Statistical Analyses

Data are presented as mean ± S.E.M. for the indicated number of experiments (*n*). Statistical significance was evaluated using the independent *t*-test or one-way ANOVA. Data were considered significant when *p* < 0.05.

## 3. Results

### 3.1. FLZ Protects INS-1E Cells and Mouse Islets from Glucotoxicity

**Figure 1:**
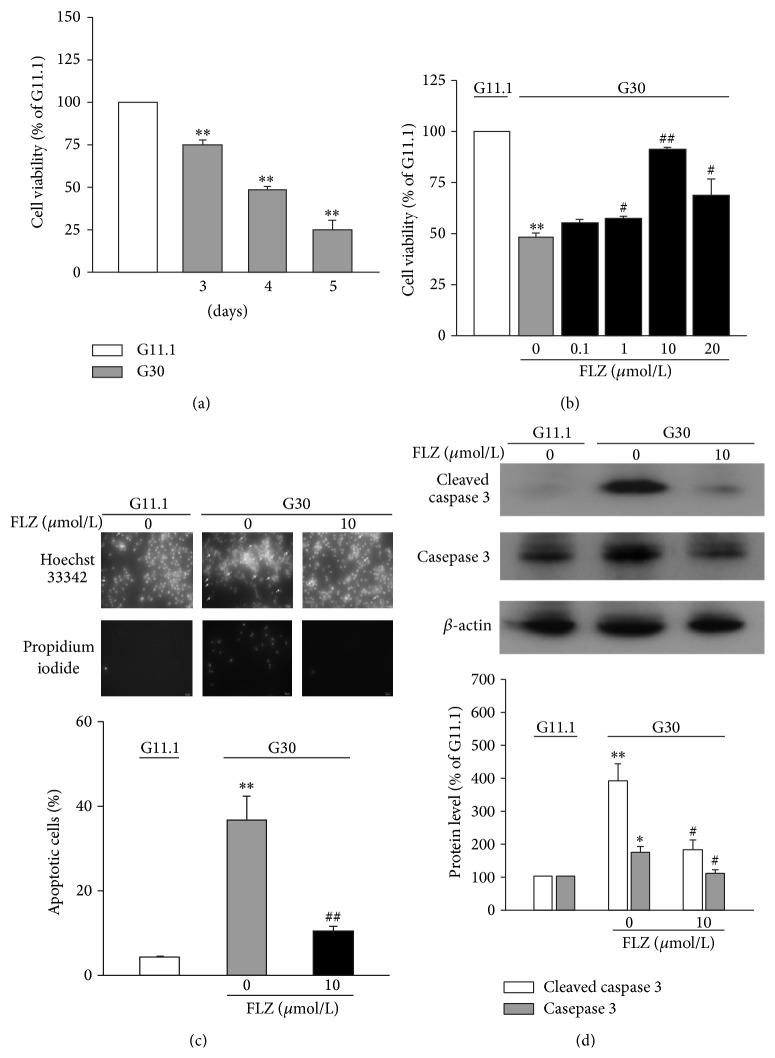
Treatment with FLZ prevents glucotoxicity in INS-1E cells. (a) INS-1E cells were cultured at G11.1 and G30 for 3–5 days and cell viability was assessed with MTT assay. Values were expressed as percentage of cell viability at G11.1. Data are means ± S.E.M. of 3–6 independent experiments. ^*∗∗*^ *p* < 0.01 versus the value at G11.1. (b) Cell viability was assessed in INS-1E cells being cultured at G11.1 and G30 for 4 days. Cells were treated without or with varying levels of FLZ. Values were expressed as percentage of cell viability at G11.1. Data are means ± S.E.M. of 3 independent experiments. ^*∗∗*^ *p* < 0.01 versus the value at G11.1; ^#^ *p* < 0.05, ^##^ *p* < 0.01 versus the value at G30 without FLZ. (c) Apoptosis assessed by HO/PI staining in INS-1E cells cultured at G11.1 and G30 for 4 days. Cells were treated without or with 10 *μ*M FLZ. Arrows indicate HO-stained cells with apoptotic bodies (upper panel). Mean values ± S.E.M. of 3 independent experiments (low panel). ^*∗∗*^ *p* < 0.01 versus the value at G11.1; ^##^ *p* < 0.01 versus the value at G30 without FLZ. (d) INS-1E cells were cultured at G11.1 or G30 for 4 days. Cells were treated without or with 10 *μ*M FLZ. Expression of total and cleaved caspase 3 protein was determined by western blot. *β*-actin was used as an internal control (upper panel). Intensities of total and cleaved caspase 3 protein expression were quantified, normalized against the level of *β*-actin, and expressed as fold of protein abundance in INS-1E cells at G11.1 (low panel). Data are means ± S.E.M. of 3 separate experiments. ^*∗*^ *p* < 0.05, ^*∗∗*^ *p* < 0.01 versus the value at G11.1; ^#^ *p* < 0.05 versus the value at G30 without FLZ.

Insulin-secreting INS-1E cells and mouse islets were cultured in 30 mM glucose (G30) for 3 to 5 days, a commonly used model for glucotoxicity [9, 10]. As shown in Figure 1(a), chronic culture of INS-1E cells in G30 resulted in marked decrease in cell viability as assayed by MTT and the value reduced by ~25% (3 days), ~52% (4 days), and ~75% (5 days) compared to that cultured in 11.1 mM glucose (G11.1), as reported previously [11]. Treatment with increasing concentrations of FLZ (0.1, 1, 10, and 20 *μ*M) reversed the effect of 30 mM glucose in a dose-dependent manner (Figure 1(b)). A concentration of 10 *μ*M of FLZ was used in most of experiments of this study, as this concentration corresponds to a maximal effective concentration reported in other studies [6]. The effect of FLZ on *β*-cell glucotoxicity was also investigated by measuring apoptotic cells as determined by HO/PI staining (Figure 1(c)). Chronic culture at G30 led to an increase in the percentages of apoptotic cells from 4 ± 0.1% to 37 ± 6% at G11.1, whereas FLZ treatment reduced the level of apoptosis to 10 ± 1% (*p* < 0.01) (Figure 1(c)). Notably, addition of FLZ essentially blocked glucotoxicity-induced increase of cleaved caspase 3 (Figure 1(d)), an activated proapoptotic protein. Thus, FLZ protects *β*-cells from glucotoxicity-induced cell death at least partly by preventing activation of caspase 3.

### 3.2. FLZ Increases Insulin Content and Secretion at Glucotoxicity

**Figure 2:**
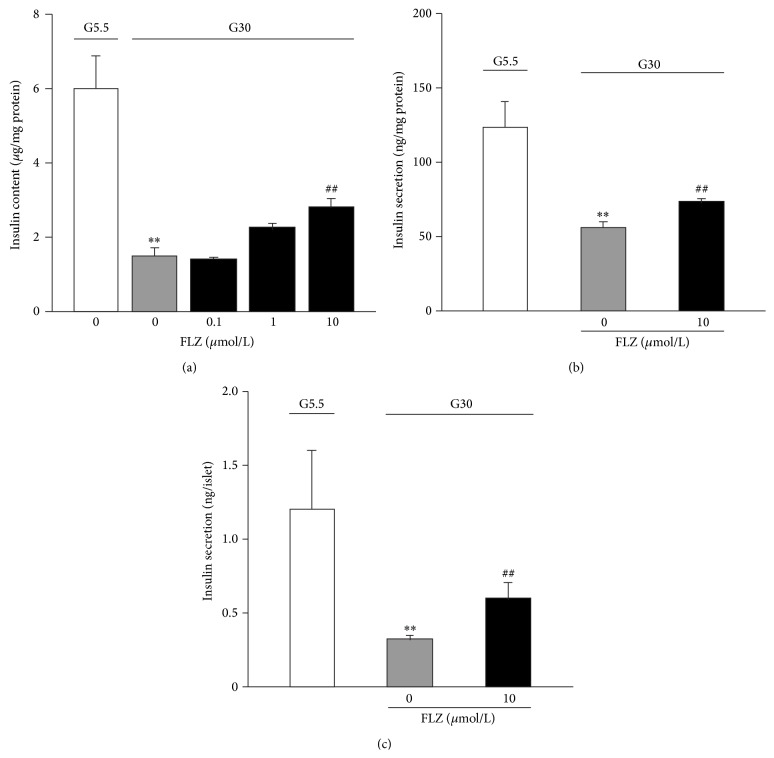
Treatment with FLZ increases insulin content and GSIS at glucotoxicity. (a) INS-1E cells were cultured at G5.5 or G30 for 3 days. Insulin content was assayed without or with varying level of FLZ. Values are normalized against total protein. Data are means ± S.E.M. of 5–8 experiments for each group. ^*∗∗*^ *p* < 0.01 versus the value at G5.5; ^##^ *p* < 0.01 versus the value at G30 without FLZ. ((b), (c)) Insulin secretion in INS-1E cells (b) or mouse islets (c) cultured at G5.5 (white bar) or G30 (grey bar) with or without 10 *μ*M FLZ (black bar) for 3 days. After preincubation with 1 mL KRB buffer for 1 h, the cells were stimulated with 1 mL KRB buffer containing 16.8 mM glucose for 30 min. The supernatants were collected for measurement of secretory insulin. Values were normalized against total protein (b) or the amount of insulin secretion per islet (c); data are means ± S.E.M. of 7–11 (b) or of 5–8 experiments (c). ^*∗∗*^ *p* < 0.01 versus the value at G5.5; ^##^ *p* < 0.01 versus the value at G30 without FLZ.

To determine whether treatment with FLZ has beneficial effects on insulin content and GSIS, INS-1E cells and mouse islets were cultured at G5.5 and G30 for 3 days. As shown in Figures 2(a)–2(c), insulin content (Figure 2(a)) and GSIS (Figures 2(b) and 2(c)) were decreased to ~25%, ~45%, and ~25%, respectively, after being cultured at G30 for 3 days, as compared to those cultured in low glucose (G5.5). In contrast, treatment with 10 *μ*M FLZ partially but significantly reversed glucotoxicity-induced reduction of insulin content dose-dependently (Figure 2(a)), as well as glucotoxicity-induced decrease of GSIS (*p* < 0.01) in INS-1E cells (Figure 2(b)) and mouse islets (Figure 2(c)).

### 3.3. FLZ Restored Glucotoxicity-Altered Expression and Nuclear Localization of PDX-1 and FOXO1

**Figure 3:**
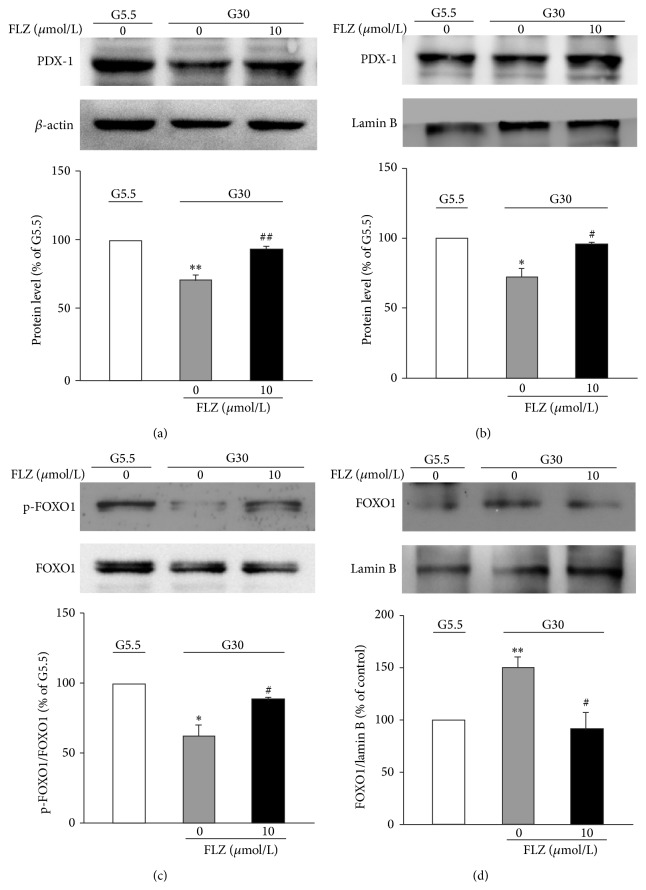
FLZ restores glucotoxicity-altered expression and intracellular localization of PDX-1 and FOXO1. ((a), (c)) INS-1E cells were cultured at G5.5 or G30 for 3 days. Cells were treated without or with 10 *μ*M FLZ. Expression of PDX-1 (a) or pFOXO1 (c) protein was determined by western blot. *β*-actin was used as an internal control ((a), upper panel). Intensities of PDX-1 (a) or pFOXO1 (c) protein expression were quantified, normalized against the level of *β*-actin (a) or total FOXO1 (c), and expressed as fold of protein abundance in INS-1E cells at G5.5 (low panels). Data are means ± S.E.M. of 3 separate experiments each. ^*∗∗*^ *p* < 0.01 versus the value at G5.5; ^##^ *p* < 0.05 versus the value at G30 without FLZ. ((b), (d)) INS-1E cells were cultured at G5.5 or G30 for 3 days. Cells were treated without or with 10 *μ*M FLZ. Nuclear protein extraction was subjected to western blot analysis. Lamin B was used as an internal control (upper panels). Intensities of PDX-1 (b) or FOXO1 (d) protein expression were quantified, normalized against the level of Lamin B, and expressed as fold of protein abundance in INS-1E cells at G5.5 (low panels). Means ± S.E.M. results of 3 separate experiments each. ^*∗*^ *p* < 0.05, ^*∗∗*^ *p* < 0.01 versus the value at G5.5; ^#^ *p* < 0.05 versus the value at G30 without FLZ.

It has been reported that glucotoxicity suppresses the expression and nuclear localization of the pancreatic and duodenal homeobox-1 (PDX-1) [12]. Consistently, our data show that chronic exposure of *β*-cells to G30 reduced PDX-1 expression (Figure 3(a)) and its nuclear localization (Figure 3(b)), accompanied by increased cytosolic distribution (see Suppl. Figure 1 in Supplementary Material available online at http://dx.doi.org/10.1155/2015/803986). In contrast, culture at G30 for 72 h resulted in reduction of phosphorylated forkhead box O1 protein (FOXO1) (Figure 3(c)) and its cytosolic distribution (Suppl. Figure 2), associated with increased nuclear distribution (Figure 3(d)). Notably, treatment with FLZ increased the PDX-1 expression and its nuclear localization (Figures 3(a) and 3(b)), accompanied by reduced cytosolic localization (Suppl. Figure 1), whereas treatment with FLZ increased phosphorylated level of FOXO1 (Figure 3(c)) and reduced its nuclear presence (Figure 3(d)), associated with its increased cytosolic localization (Suppl. Figure 2).

### 3.4. The Antiglucotoxic Effect of FLZ Is Mediated via Akt

**Figure 4:**
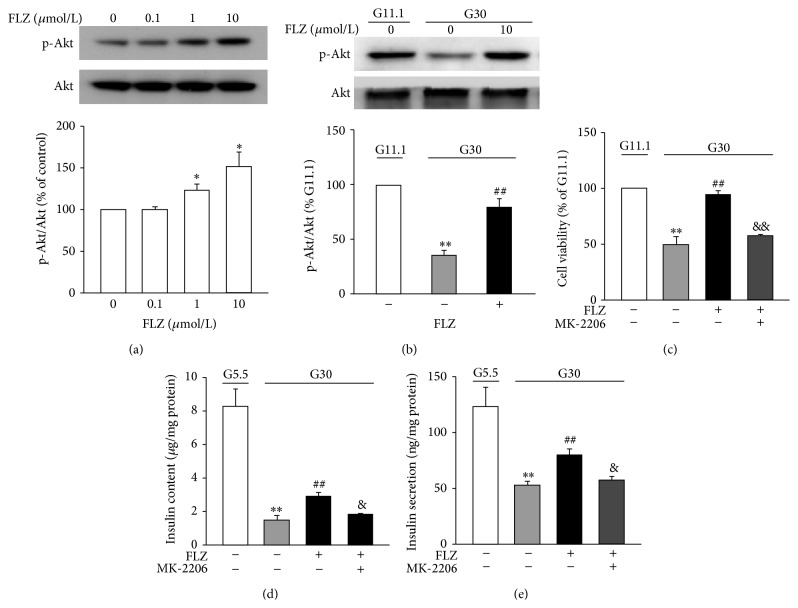
FLZ acts via stimulation of Akt. (a) INS-1E cells were treated with 0.1 *μ*M, 1 *μ*M, and 10 *μ*M FLZ for 8 h. Expression of p-Akt and total Akt protein was determined by western blot. Mean ± S.E.M. of three separate experiments. Intensities of p-Akt protein expression were quantified, normalized against the level of total Akt, and expressed as fold of protein abundance in INS-1E cells treated with DMSO. ^*∗*^ *p* < 0.05 versus control. (b) INS-1E cells were cultured at G11.1 or G30 for 4 days. Cells were treated without or with 10 *μ*M FLZ. Expression of p-Akt and total Akt protein was determined by western blot. Mean ± S.E.M. of seven separate experiments. ^*∗∗*^ *p* < 0.01 versus the value at G11.1; ^##^ *p* < 0.01 versus the value at G30 without FLZ. (c) Cell viability was assessed in INS-1E cells being cultured at G11.1 and G30 for 4 days. Cells were treated without or with 10 *μ*M FLZ or simultaneous addition of 10 *μ*M FLZ and 50 nM MK-2206. Values were expressed as percentage of cell viability at G11.1. Data are means ± S.E.M. of 3-4 independent experiments. ^*∗∗*^ *p* < 0.01 versus the value at G5.5; ^##^ *p* < 0.01 versus the value at G30 without FLZ; ^&&^ *p* < 0.01 versus the value at G30 with FLZ. (d) Insulin content was assayed without or with 10 *μ*M FLZ or simultaneous addition of 10 *μ*M FLZ and 50 nM MK-2206. Values are normalized against total protein. Data are means ± S.E.M. of 4–12 experiments. ^*∗∗*^ *p* < 0.01 versus the value at G5.5; ^##^ *p* < 0.01 versus the value at G30 without FLZ; ^&^ *p* < 0.05 versus the value at G30 with FLZ. (e) Insulin secretion was assayed in INS-1E cells treated without or with 10 *μ*M FLZ or simultaneous addition of 10 *μ*M FLZ and 50 nM MK-2206. Values were normalized against total protein. Data are mean ± S.E.M. of 5–16 separate experiments. ^*∗∗*^ *p* < 0.01 versus the value at G5.5; ^##^ *p* < 0.01 versus the value at G30 without FLZ; ^&^ *p* < 0.05 versus the value at G30 with FLZ.

Akt is an important prosurvival kinase. We next explored whether the antiglucotoxic effect of FLZ is involved in regulating activation of Akt. The results showed that FLZ increased the phosphorylation of Akt (p-Akt) in a dose-dependent manner (Figure 4(a)). As reported in another study [13], incubation of cells at G30 for 4 days essentially reduced the level of phosphorylated Akt, as compared to that at G11.1. It is of note that treatment with 10 *μ*M FLZ significantly increased the level of p-Akt (*p* < 0.01) (Figure 4(b)). In the presence of the Akt inhibitor MK-2206 (50 nM), which has no effect on G30 condition (Suppl. Figure 4), FLZ lost its ability to reduce the effects of glucotoxicity on cell viability (Figure 4(c)), insulin content (Figure 4(d)), and GSIS (Figure 4(e)). In addition, the Akt-mediated effect of FLZ was further confirmed by the data showing that the effect of FLZ on PDX-1 was completely abolished by MK-2206 (Suppl. Figures 3A–3C).

**Figure 5:**
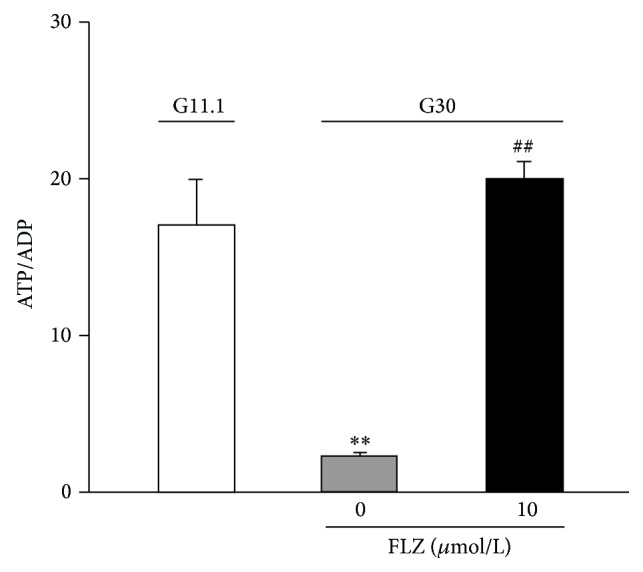
FLZ increases cytosolic ATP/ADP ratio at glucotoxicity. INS-1E cells were cultured at G11.1 or G30 for 3 days. Cells were treated without or with 10 *μ*M FLZ. Data are mean ± S.E.M. of seven independent experiments. ^*∗∗*^ *p* < 0.01 versus the value at G5.5; ^##^ *p* < 0.01 versus the value at G30 without FLZ.

Based on the observations that FLZ protected dopaminergic neurons against apoptosis via reducing mitochondrial oxidative stress [6], we next explored whether the fact that FLZ prevents glucotoxicity-induced *β*-cell failure would be due to improvement of mitochondrial function. INS-1E cells were cultured at G11.1 and G30 mM for 4 days and treated without or with 10 *μ*M FLZ. As shown in Figure 5, chronic culture at G30 substantially reduced the level of ATP/ADP ratio, consistent with previous reports [8]. Treatment with FLZ significantly increased ATP/ADP ratio.

## 4. Discussion

In the present study, we provide evidence that the synthetic derivative of squamosamide FLZ prevents glucotoxicity-induced *β*-cell dysfunction by a mechanism involving Akt and its downstream target FOXO1.

FLZ is a synthetic derivative of squamosamide from a Chinese herb and has been found to be capable of protecting dopaminergic cells against MPP^+^- [2, 3] and 6-OHDA- [4, 5] induced apoptosis, via activation of PI3K/Akt signaling pathway [2, 6]. Like its effects found in dopaminergic neurons, FLZ protects *β*-cells against glucotoxicity-induced apoptosis through preventing activation of caspase 3 induced by glucotoxicity (Figure 1(d)) and thus increases cell viability in a dose-dependent manner (Figures 1(a)–1(c)). In addition, we also show that treatment with FLZ significantly decreases glucotoxicity-induced reduction of insulin content (Figure 2(a)), as well as glucose-stimulated insulin secretion in INS-1E cells and mouse islets (Figures 2(b) and 2(c)). Given that reduced *β*-cell mass and GSIS by chronic hyperglycemia are essential for development of T2D [14], this antiglucotoxic effect of FLZ is of vital merit for treatment of T2D.

The pancreatic and duodenal homeobox factor-1 (PDX-1) plays a crucial role in maintaining mature *β*-cell function, such as transactivating the insulin gene and the genes controlling GLUT2 and glucokinase that are important for glucose sensing and metabolism [15, 16]. Indeed, heterozygous mutation of PDX-1 causes glucose intolerance, associated with increased islet apoptosis and impaired GSIS, which collectively results in maturity onset diabetes [16, 17]. In pancreatic *β*-cells, PDX-1 is negatively regulated by FOXO1 [18]; the latter acts as a transcriptional brake on PDX-1 [19]. Moreover, FOXO1 and PDX-1 are mutually exclusive patterns of nuclear location in *β*-cells [19]. Thus, FOXO1 inactivation leads to FOXO1 nuclear exclusion and PDX-1 expression and finally *β*-cell proliferation and insulin gene expression [19, 20]. Our data extend the previous observations by showing that chronic exposure of *β*-cells to elevated glucose stimulates FOXO1 activation and increases its nuclear location (Figures 3(c) and 3(d)) and therefore results in reduced expression and nuclear location of PDX-1 (Figures 3(a) and 3(b)) and finally reduction of insulin content, *β*-cell mass, and insulin secretion. However, treatment with FLZ reverses the detrimental effect of glucotoxicity on PDX-1. Therefore, these data suggest that FLZ improves *β*-cell mass and function via regulation of FOXO1 and PDX-1 pathway.

In addition, it is known that pancreatic *β*-cells convert glucose to ATP and thereby lead to increase of the cellular ATP/ADP ratio and promote the closure of the ATP-sensitive, potassium channels (K^+^_ATP_ channels) which is the initial triggering event for insulin secretion [21]. Therefore, the effect of FLZ on ATP/ADP ratio (Figure 5) may also be involved in its improvement in insulin secretion in *β*-cells at glucotoxicity.

It is known that FOXO1 is inhibited by Akt upon phosphorylation [19]. Activation of Akt-FOXO1 results in nuclear exclusion of FOXO1 and subsequently induces transcription of PDX-1 [19]. Akt/PKB is an important prosurvival kinase and is essential for maintaining *β*-cell mass and function. Our data reveal for the first time that FLZ prevents glucotoxicity-induced *β*-cell failure, an effect via activation of Akt. Three pieces of evidence corroborate this notion. First, FLZ stimulated Akt activation (Figures 4(a) and 4(b)). Second, treatment of INS-1E cells with Akt inhibitor MK-2206 almost abolished FLZ increased cell viability (Figure 4(c)), insulin content (Figure 4(d)), and GSIS (Figure 4(e)). Third, while FLZ was capable of reversing glucotoxicity-altered expression and nuclear location of PDX-1 (Figures 3(a) and 3(b)), FLZ failed to do so in the presence of Akt inhibitor MK-2206 (Suppl. Figures 3A and 3B).

The detailed mechanisms as to how FLZ leads to phosphorylation of Akt require future investigation. However, our results imply that the FLZ-induced Akt activation may be attributed to its ability of restoring mitochondrial function impaired by glucotoxicity, given FLZ increased cytosolic ATP/ADP ratio diminished by glucotoxicity (Figure 5). This notion is in fact supported by the observation that blocking mitochondrial fission with subsequent reduction in H_2_O_2_ increased insulin-induced Akt/PKB phosphorylation [22].

In conclusion, our data demonstrate that FLZ prevents glucotoxicity-induced *β*-cell dysfunction. The mechanism involved could imply Akt activation and sequentially phosphorylates FOXO1 and leads to its nuclear exclusion and consequently upregulates transcription of PDX-1 [19]. This cascade of events will finally result in increased insulin production and *β*-cell mass. Taken together, our results suggest that FLZ could serve as a potential therapeutic agent for the treatment of hyperglycemia-induced *β*-cell failure.

## Supplementary Material

Supplementary data showed that FLZ decreased PDX-1 cytosolic localization (Suppl.Fig.1), in contrast, it increased FOXO1 cytosolic localization (Suppl.Fig.2). MK-2206 inhibited PDX-1 expression and nuclear localization induced by FLZ (Suppl.Fig.3). MK-2206 had no effect on cell viability, insulin content and secretion in INS-1E cells at G30 (Suppl.Fig.4).
