# Supplementary material for: Squamosamide Derivative FLZ Protects Pancreatic β-Cells from Glucotoxicity by Stimulating Akt-FOXO1 Pathway
Source: J Diabetes Res. 2015 Jun 17;2015:803986. doi: 10.1155/2015/803986 (PMC4488173; doi:10.1155/2015/803986)
Supplement: Supplementary file 1 — Supplementary data showed that FLZ decreased PDX-1 cytosolic localization (Suppl.Fig.1), in contrast, it increased FOXO1 cytosolic localization (Suppl.Fig.2). MK-2206 inhibited PDX-1 expression and nuclear localization induced by FLZ (Suppl.Fig.3). MK-2206 had no effect on cell viability, insulin content and secretion in INS-1E cells at G30 (Suppl.Fig.4). [file 803986.f1.pdf]

Supplementary materials: Kong et al.

Figure legends

Suppl.Fig.1 FLZ decreased PDX-1 cytosolic localization.

INS-1E were cultured at G5.5 or G30 in the presence or absence of 10 μM FLZ for 3 days. Then cytosolic and nuclear protein were extracted and subjected to western blot analysis. Values represent mean±SEM (n=3).**, p<0.01 vs. cells at G5.5; #, p<0.05 vs. cells at G30 without FLZ.

Suppl.Fig.2 FLZ increased FOXO1 cytosolic localization.

INS-1E were cultured at G5.5 or G30 with or without 10 μM FLZ for 3 days. Then cytosolic and nuclear protein were extracted and subjected to western blot analysis. Values represent mean±SEM (n=3). **, p<0.01 vs. cells cultured in G5.5; #, p<0.05 vs. cells cultured in G30 without FLZ .

Suppl.Fig.3 MK-2206 inhibited PDX-1 expression and nuclear localization induced by FLZ.

(A) INS-1E were cultured at G5.5 or G30 with or without FLZ or MK-2206 for 3 days, whole cell proteins were extracted and were subjected to western blot analysis. Values represent mean±SEM (n=3). **, p<0.01 vs. cells at G5.5; ##, p<0.01 vs. cells at G30 without FLZ; &, p<0.05 vs. cells at G30 with FLZ .

(B) and **(C)** INS-1E were cultured at G5.5 or G30 with or without FLZ or MK-2206 for 3 days. Cytosolic and nuclear protein were extracted and subjected to western blot

analysis. Values represent mean±SEM (n=3). **, p<0.01 vs. cells at G5.5; #, p<0.05, ##, p<0.01 vs. cells at G30 without FLZ; &, p<0.05 vs. cells at G30 with FLZ.

Suppl.Fig.4 Effect of MK-2206 on cell viability, insulin content and secretion in INS-1E cells at G30.

(A) INS-1E cells were cultured at G30 with or without 50 nM MK-2206 for 4 days. Cell viability was assessed with MTT assay. Data are means ± S.E.M of 3 independent experiments.

(B, C) INS-1E cells were cultured at G30 in the presence or absence of 50 nM MK-2206 for 3 days. Then investigated insulin content **(B)** or insulin secretion **(C)**. Values were normalized against total protein**.** Data are means ± S.E.M of 3 experiments **.**

Suppl.Fig.1

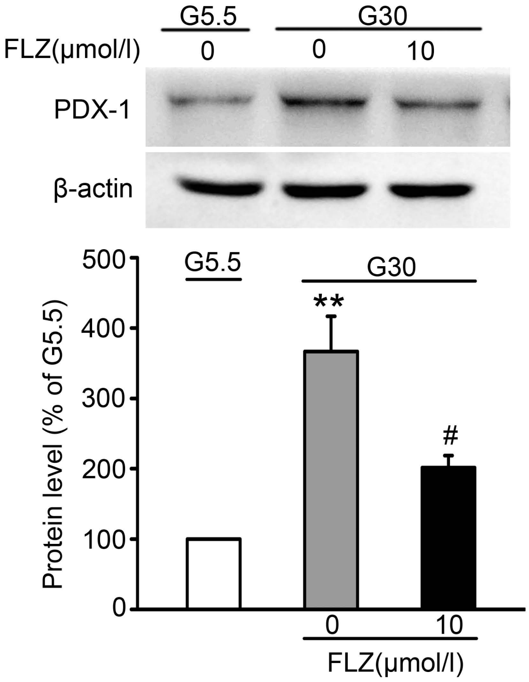
G5.5
G30
FLZ(μmol/l)
0
0
10
PDX-1
β-actin
500
400
300
200
100
0
Protein level (% of G5.5)
G5.5
G30
**
#
0
10
FLZ(μmol/l)

Suppl.Fig.2

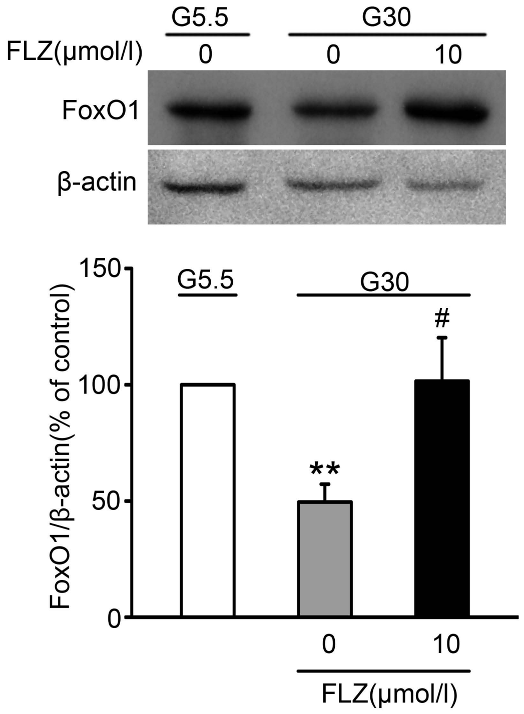
G5.5
G30
FLZ(μmol/l)
0
0
10
FoxO1
β-actin
150
100
50
0
G5.5
G30
#
**
FoxO1/β-actin(% of control)
0
10
FLZ(μmol/l)

Suppl.Fig.3

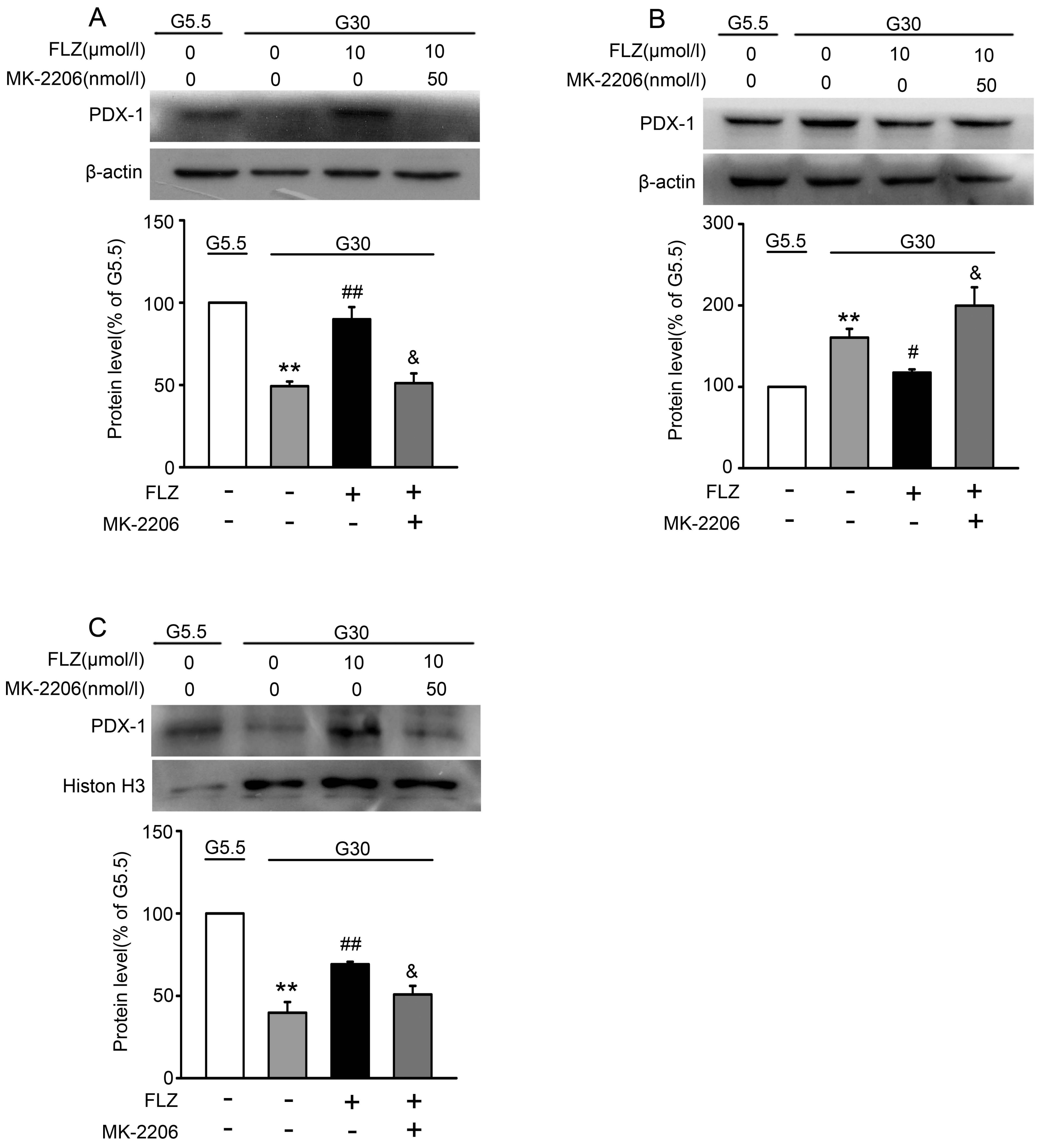
A
G5.5
G30
FLZ(μmol/l) 0 0 10 10
MK-2206(nmol/l) 0 0 0 50
PDX-1
β-actin
Protein level(% of G5.5)
150
100
50
0
**
##
&
FLZ - - + +
MK-2206 - - - +
B
G5.5
G30
FLZ(μmol/l) 0 0 10 10
MK-2206(nmol/l) 0 0 0 50
PDX-1
β-actin
Protein level(% of G5.5)
300
200
100
0
**
#
&
FLZ - - + +
MK-2206 - - - +
C
G5.5
G30
FLZ(μmol/l) 0 0 10 10
MK-2206(nmol/l) 0 0 0 50
PDX-1
Histon H3
Protein level(% of G5.5)
150
100
50
0
**
##
&
FLZ - - + +
MK-2206 - - - +

Suppl.Fig.4

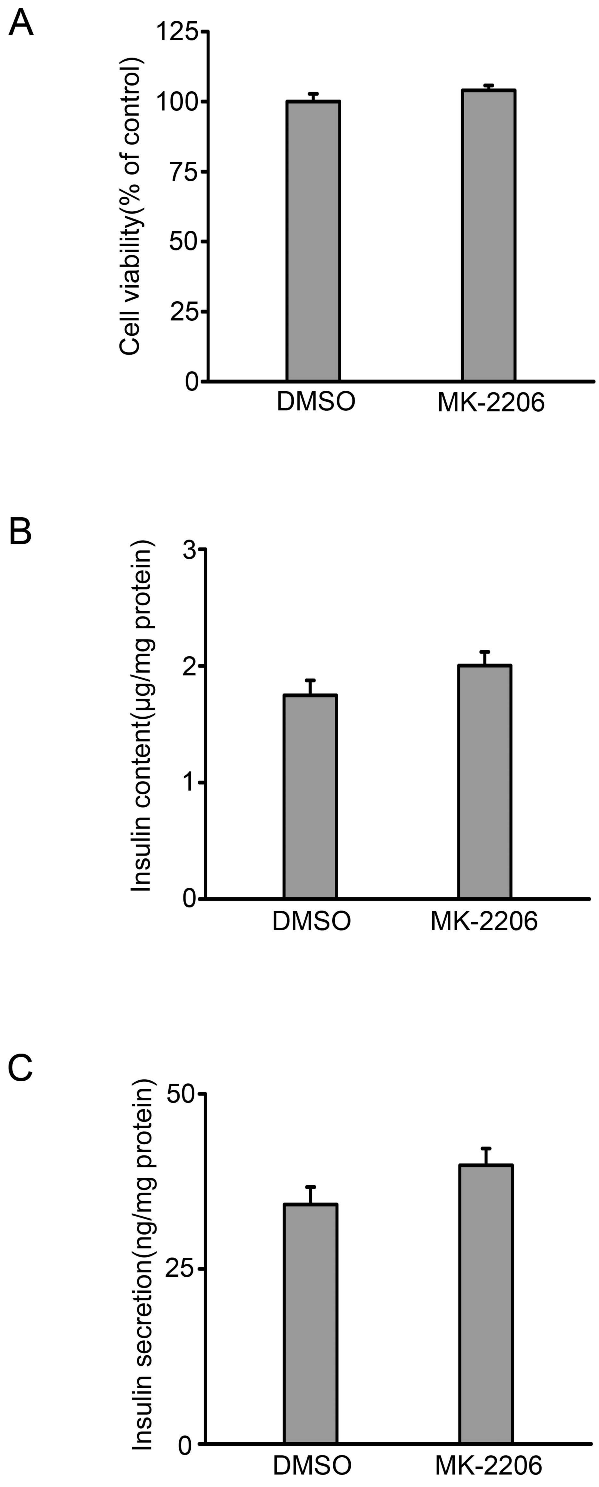
A
125
100
75
50
25
0
Cell viability(% of control)
DMSO
MK-2206
B
3
2
1
0
Insulin content(µg/mg protein)
DMSO
MK-2206
C
50
25
0
Insulin secretion(ng/mg protein)
DMSO
MK-2206
